# Classification of forensically-relevant larvae according to instar in a closely related species of carrion beetles (Coleoptera: Silphidae: Silphinae)

**DOI:** 10.1007/s12024-016-9774-0

**Published:** 2016-04-12

**Authors:** Katarzyna Frątczak, Szymon Matuszewski

**Affiliations:** Laboratory of Criminalistics, Adam Mickiewicz University, Św. Marcin 90, 61-809 Poznan, Poland; Department of Animal Taxonomy and Ecology, Adam Mickiewicz University, Umultowska 89, 61-614 Poznan, Poland

**Keywords:** Forensic entomology, Instar determination, *Thanatophilus sinuatus*, *Thanatophilus rugosus*, *Necrodes littoralis*, *Oiceoptoma thoracicum*

## Abstract

Carrion beetle larvae of *Necrodes littoralis* (Linnaeus, 1758), *Oiceoptoma thoracicum* (Linnaeus, 1758), *Thanatophilus sinuatus* (Fabricius, 1775), and *Thanatophilus rugosus* (Linnaeus, 1758) (Silphidae: Silphinae) were studied to test the concept that a classifier of the subfamily level may be successfully used to classify larvae according to instar. Classifiers were created and validated using a linear discriminant analysis (LDA). LDA generates classification functions which are used to calculate classification values for tested specimens. The largest value indicates the larval instar to which the specimen should be assigned. Distance between dorsal stemmata and width of the pronotum were used as classification features. The classifier correctly classified larvae of *N. littoralis* and *O. thoracicum*, whereas in the case of *T. sinuatus* and *T. rugosus* a few misclassifications were recorded. For this reason, a separate genus level classifier was created for larvae of *Thanatophilus*. We conclude that larval instar classifiers of the subfamily or genus level have very high classification accuracy and therefore they may be safely used to classify carrion beetle larvae according to instar in forensic practice.

## Introduction

Age determination of beetle larvae is a difficult task [1]. It can be estimated from length, weight, or developmental stage [2–4]. Under controlled laboratory conditions, when insects may be continuingly monitored, it is usually beyond doubt what stage of development specimen represents, as ecdysis is confirmed by the presence of exuvia [5]. In the practice of forensic entomology developmental stage of an immature insect sampled from a body has to be classified according to instar using different methods. In the case of forensically important flies, instar classification is easy due to robust qualitative diagnostic features [6, 7]. For most beetles, classification of larvae according to instar is a problem, as there are no morphological, qualitative features specific for particular instars [7]. Measurements of larvae were found to be useful for this purpose and several quantitative features were proposed for the Palaearctic species *Necrodes littoralis*, *Creophilus maxillosus* (Linnaeus, 1758) [7], and *Sciodrepoides watsoni* (Spence, 1815) [8], a Neotropical species *Oxelytrum discicolle* (Brullé, 1840) [1], and the Nearctic species *Necrodes surinamensis* (Fabricius, 1775), *Necrophila americana* (Linnaeus, 1758), and *Oiceoptoma inaequale* (Fabricius, 1781) [9]. This approach is based mainly on the ranges of features characteristic of particular instars [1, 8, 9]. Moreover, some authors used statistical methods to identify features which are useful for instar classification [1, 7, 9]. Recently, linear discriminant analysis was used to generate classification functions, on the basis of which larval specimens may be classified according to instar from measurements of just two features [7].

Some closely related beetle taxa, particularly species of the same genus or subfamily, have similar larval sizes or at least similar sizes of some larval structures, e.g. species of *Dermestes*, *Omosita*, or Silphinae [10–12]. Accordingly, we predict that larvae of some species may be classified according to instar using general classifiers instead of species level classifiers. This prediction is supported by a recent study in which a classifier for larval Carabidae had a correct classification rate of 71 % [13]. Because general classifiers would be highly useful for forensic entomologists, in this article we test the concept of a subfamily level classifier in the case of forensically significant carrion beetles (Silphidae: Silphinae). The most common and forensically important species of Silphinae in Europe are *Thanatophilus sinuatus*, *Thanatophilus rugosus*, *Necrodes littoralis*, and *Oiceoptoma thoracicum* [14–18]. They were regularly and abundantly sampled from pig carcasses in many forensic carrion studies [14–16, 19–24]. In the current study these species were used to test the concept that a classifier of the subfamily level may be successfully used to classify larvae according to instar.

## Materials and methods

### Larvae

Adult *Thanatophilus sinuatus* and *Thanatophilus rugosus* were collected from 6 rabbit carcasses exposed in grasslands (52°310′N, 16°540′E; Western Poland, Central Europe). Carcasses were purchased in a pet store. Three carcasses were exposed on 3 July 2014 and adult *T. sinuatus* were collected on 8 July 2014. Another three carcasses were exposed on 15 May 2015 and adult *T. rugosus* were collected on 23 May 2015. Laboratory colonies consisted of 45 specimens (25 females, 20 males) in the case of *T. sinuatus* and 37 specimens (21 females, 16 males) in the case of *T. rugosus*. Beetles were fed with pork. Because we wanted to get larvae representing the full range of variation in size, specimens were sampled just after ecdysis and fully sclerotized. Larvae were killed and kept in 70 % ethanol.

Larvae of *Oiceoptoma thoracicum* were collected from pig carcasses exposed in alder forest on 18 April 2011 during our earlier decomposition studies. Specimens were preserved in 70 % ethanol. Special care was taken to choose larvae representing the full range of variation in size. In each larval stage we chose fully sclerotized specimens as well as creamy-white larvae shortly after ecdysis. For *Necrodes littoralis* it was decided to use measurement data from our previous study [7].

A subfamily level classifier was created using 60 training larvae (20 per instar) of each species. The classifier was tested with training larvae and 30 test larvae (10 per instar) of each species.

### Measurements

**Fig. 1:**
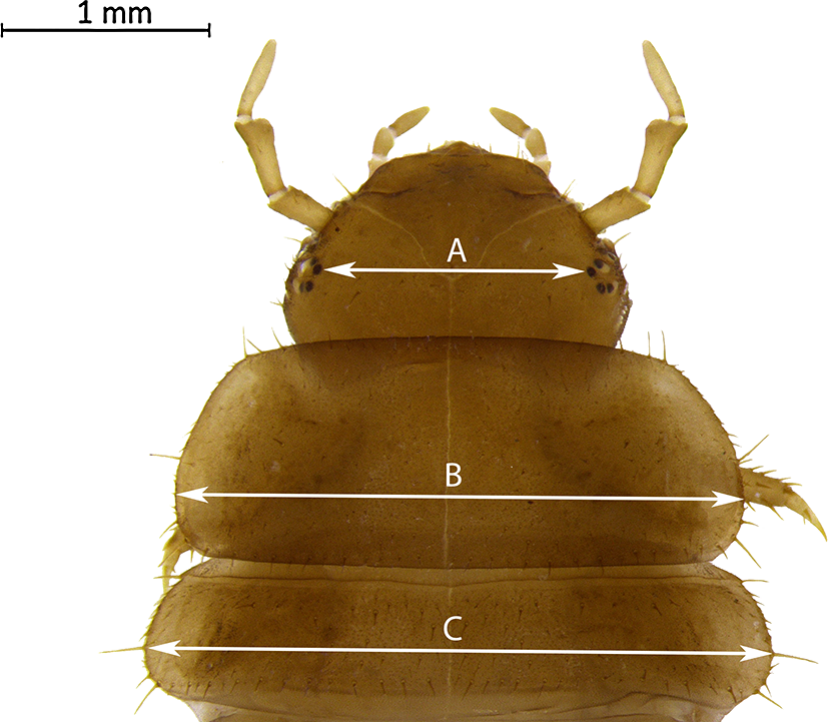
Second instar larva of *T. sinuatus*: *A* distance between dorsal stemmata, *B* width of the pronotum, *C* width of the mesonotum

30 larvae (20 training larvae and 10 test larvae) of each instar and species were measured according to the following features: distance between dorsal stemmata (hereafter “stemmata”), width of the pronotum (hereafter “pronotum”), and width of the mesonotum (hereafter “mesonotum”) (Fig. 1). These features were selected due to their good performance in previous, similar studies [1, 7, 9] and similar ranges in particular species [7, 11, 12]. Measurements were taken with Leica Application Suite 4.1 from digital photographs made with a Leica M165C stereomicroscope and a Leica DFC450 camera. Specimens were deposited at the Laboratory of Criminalistics (Adam Mickiewicz University, Poznań, Poland).

### Data analyses

Cluster analysis was used to confirm the number of larval stages in *O. thoracicum*. Classifiers were created and validated using a linear discriminant analysis (LDA). The analysis generates classification functions which may be used to classify larvae according to instar with the following formula:

$$ S_{x} = c + w_{1} \times f_{1} + w_{2} \times f_{2} + \cdots + \, w_{i} \times f_{i} ; $$

where *S*_*x*_ is the classification value for an instar *x*, *c* is the constant, *w* represents the weight for a measured feature, and *f* is the measurement of a feature. Three such formulas are created, one per instar. In order to classify a specimen sampled from a cadaver, its measurements are used to calculate classification values with each formula and the largest value indicates the larval instar to which the specimen belongs.

Moreover, descriptive statistics (means, ranges, and coefficients of variation) were calculated for all features. A level of 5 % significance was accepted in all analyses. Calculations were made using STATISTICA 10 (StatSoft, Inc. 2011).

## Results

Cluster analysis revealed three size clusters in *O. thoracicum* larvae confirming the presence of three larval stages.

**Fig. 2:**
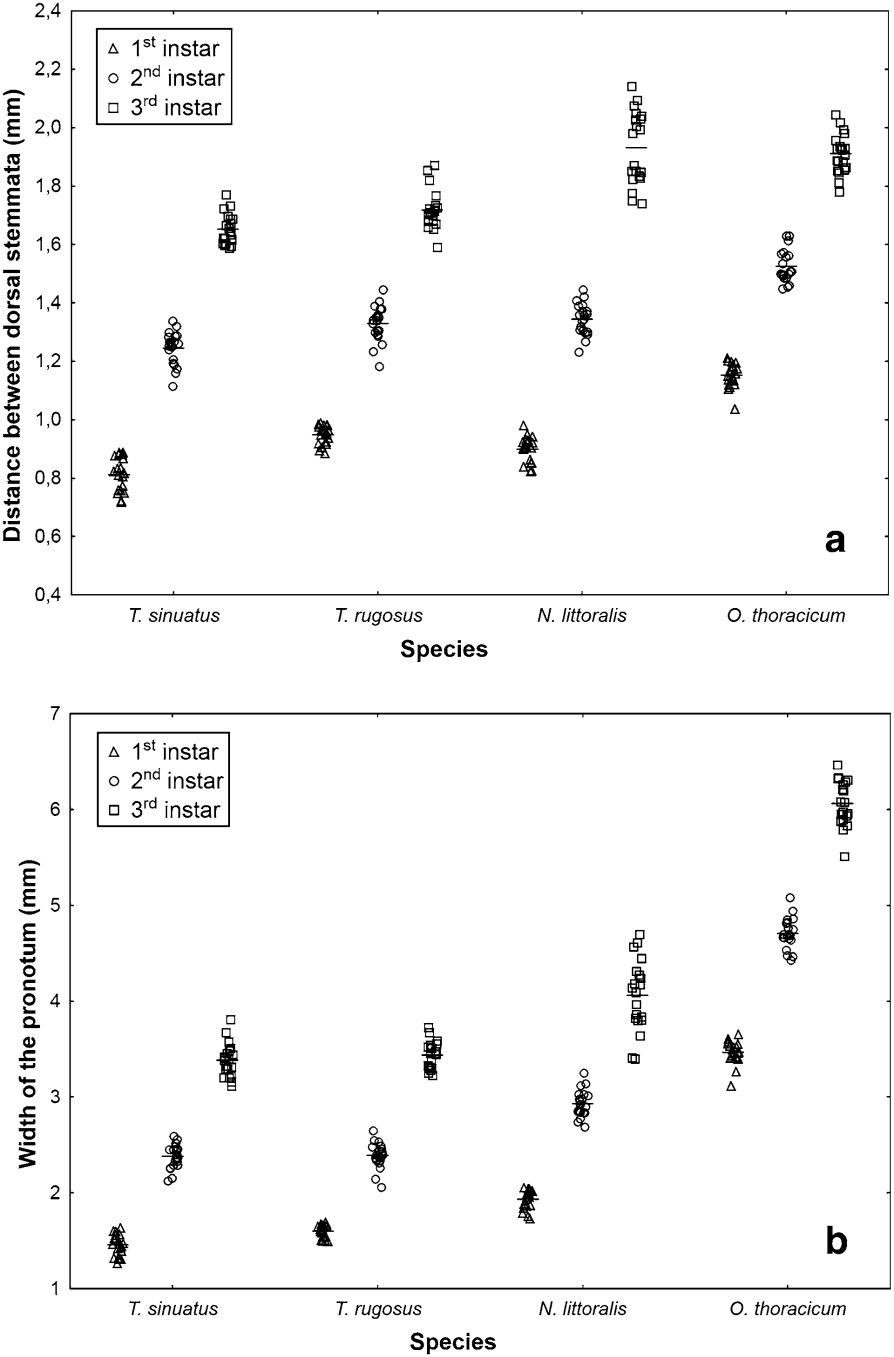
Measurements of distance between dorsal stemmata (**a**) and width of the pronotum (**b**) in larval stages of *T. sinuatus*, *T. rugosus*, *N. littoralis*, and *O. thoracicum* (training larvae); *symbols* raw data, *solid line* mean

The distance between dorsal stemmata and the width of the pronotum were used to create a classifier (Fig. 2; Table 1). The width of the mesonotum was excluded due to its almost perfect correlation with the width of the pronotum. The model incorporated both features (Table 1) with the highest contribution of distance between dorsal stemmata. The first discriminant function explained approximately 99 % of the variance. Validation with training larvae gave almost perfect results. Only one second instar larva was misclassified as a first instar larva. Validation with test larvae gave perfect results in the case of *N. littoralis* and *O. thoracicum*. A few misclassifications were, however, observed in the case of *T. sinuatus* and *T. rugosus* (Tables 2, 3).

**Table 1:** Classification functions in the subfamily level classifier for larval Silphinae

Feature	Larval instar		
	I	II	III
Stemmata	110.1322	155.9285	204.461
Pronotum	−9.5795	−13.4486	−17.456
Constant	−43.4476	−86.3388	−148.4

**Table 2:** The classification matrix for test larvae of *T. sinuatus*

	1st instar	2nd instar	3rd instar	Percentage correct
1st instar	10	0	0	100
2nd instar	0	10	0	100
3rd instar	0	3	7	70
Total	10	13	7	90

**Table 3:** The classification matrix for test larvae of *T. rugosus*

	1st instar	2nd instar	3rd instar	Percentage correct
1st instar	10	0	0	100
2nd instar	0	10	0	100
3rd instar	0	1	9	90
Total	10	11	9	96.7

Because classifiers created for forensic purposes should be more accurate, it was decided to generate a genus level classifier for *Thanatophilus* larvae (Table 4). Its validation with training and test larvae was 100 % correct.

**Table 4:** Classification functions in the genus level classifier for larval *Thanatophilus*

Feature	Larval instar		
	I	II	III
Stemmata	105.5746	137.978	148.949
Pronotum	45.3618	80.206	131.842
Constant	−82.2481	−185.553	−351.543

## Discussion

High classification accuracy of the subfamily level classifier prompted us to recommend that in forensic practice all larvae of *N. littoralis* and *O. thoracicum*, as well as the fully sclerotized larvae of *Thanatophilus*, are to be classified according to instar with the simple subfamily level classifier. For the *Thanatophilus* larvae just after ecdysis it is suggested the genus level classifier is used, due to its higher accuracy.

The accuracy of the classifier created for the family Carabidae was approximately 71 % [13]. Its poor performance indicates that family level classifiers will have a low accuracy for classification. Moreover, it seems that variation of larval size in families or higher taxa is so large that classifiers for such taxa are very likely to have low accuracy. Because in a forensic context low accuracy is unacceptable, classifiers of the genus or subfamily level should be created and current results demonstrate that they may classify forensic specimens according to instar with very high accuracy.

Very high classification accuracy—as currently recorded in the subfamily Silphinae—results mostly from similar larval sizes in the species used and their consistent changes across larval stages (Table 5). These changes are the most evident in the case of highly sclerotized structures, so not surprisingly the most useful features in this respect were the distance between the dorsal stemmata [1, 7, 9], the width of the pronotum [1, 7, 9], the width of the mesonotum [7], or the width of the head [8]. Many other forensically relevant genera or subfamilies of beetles have uniform larval sizes. Accordingly, future studies should seek general classifiers in such taxa as *Necrobia*, *Omosita*, or *Dermestes*. In these taxa head structure sizes seem to be the most promising for distinguishing larval stages, as the rest of their body is not highly sclerotized. Interestingly, in the case of *Dermestes* larvae the number of instars may vary depending on several factors [25]. Because such variation may be a problem for classification, resultant limitations need to be identified.

**Table 5:** Means and ranges for features of larval *T. sinuatus*, *T. rugosus*, *N. littoralis*, and *O. thoracicum* (combined data for training and test larvae)

Species	Instar	Feature								
		Stemmata			Pronotum			Mesonotum		
		Mean (mm)	Range (mm)	CV (%)	Mean (mm)	Range (mm)	CV (%)	Mean (mm)	Range (mm)	CV (%)
*Thanatophilus sinuatus*	I	0.821	0.72–0.89	6.3	1.486	1.26–1.63	6.872	1.59	1.32–1.78	7.407
	II	1.231	1.12–1.34	4.808	2.358	2.11–2.59	5.642	2.553	2.29–2.85	5.798
	III	1.635	1.4–1.77	4.776	3.381	2.91–3.81	6.08	3.716	3.14–4.17	6.929
*Thanatophilus rugosus*	I	0.943	0.88–0.99	3.101	1.592	1.49–1.74	4.395	1.718	1.58–1.86	5.145
	II	1.33	1.18–1.45	4.433	2.406	2.06–2.68	5.843	2.595	2.28–2.86	5.34
	III	1.711	1.54–1.87	4.272	3.425	3.12–3.73	4.195	3.795	3.47–4.18	4.658
*Necrodes littoralis*	I	0.896	0.82–0.98	4.614	1.929	1.73–2.05	4.989	2.057	1.84–2.22	4.668
	II	1.339	1.23–1.45	4.122	2.923	2.65–3.25	4.615	3.003	2.68–3.34	5.837
	III	1.89	1.74–2.14	6.337	3.936	3.312–4.7	9.432	4.474	3.96–6.67	6.668
*Oiceoptoma thoracicum*	I	1.155	1.04–1.25	3.571	3.507	3.12–3.75	3.765	3.895	3.53–4.18	3.596
	II	1.53	1.45–1.63	3.164	4.715	4.43–5.08	3.151	5.269	5.03–5.72	3.011
	III	1.917	1.78–2.05	3.085	6.042	5.51–6.47	3.523	6.789	6.22–7.18	3.229

*CV* coefficient of variation

The current approach may also be used in the case of some Diptera species. Although most forensically significant Diptera have qualitative morphological features useful for instar determination, there are some species in which classification of larvae according to instar is a problem, e.g. *Hermetia illucens* (Linnaeus, 1758) (Stratiomyidae). This species has six larval stages and no clear morphological feature that is useful for their differentiation [26].

In order to create classifiers, we used laboratory-reared larvae (*T. sinuatus* and *T. rugosus*) as well as larvae from decomposition studies (*N. littoralis*, *O. thoracicum*). For future research it is recommended that only laboratory-reared larvae are used, as such material facilitates the selection of larvae representing a full range of variation in size.

In conclusion, current results demonstrate that larval instar classifiers of the subfamily or genus level have a very high accuracy of classification in the case of carrion beetles (Silphidae: Silphinae). For this reason they may be recommended as useful for forensic practice.

## Key points

1. Larval instar classifiers of the subfamily and genus level were tested in the case of forensically important species of carrion beetles (Silphidae: Silphinae).
2. Measurements of the distance between the dorsal stemmata and the width of the pronotum were found to be useful for instar determination.
3. A subfamily level classifier correctly classified all larvae of *N. littoralis* and *O. thoracicum*, as well as the fully sclerotized larvae of *Thanatophilus*.
4. For the *Thanatophilus* larvae just after ecdysis it is suggested to use the genus level classifier.
